# Development and Verification of a Prostate Cancer Prognostic Signature Based on an Immunogenomic Landscape Analysis

**DOI:** 10.3389/fonc.2021.711258

**Published:** 2021-09-09

**Authors:** Hong Cheng, Yi Wang, Chunhui Liu, Tiange Wu, Shuqiu Chen, Ming Chen

**Affiliations:** ^1^Department of Urology, Zhongda Hospital Affiliated to Southestern China University, Nanjing, China; ^2^Department of Urology, Affiliated Hospital of Nantong University, Nantong, China

**Keywords:** TCGA, prostate cancer, prognostic signature, tumor immunology, S100A2

## Abstract

**Purpose:**

Prostate cancer (PCa) has a high incidence among older men. Until now, there are no immunological markers available to predict PCa patients’ survival. Therefore, it is necessary to explore the immunological characteristics of PCa.

**Methods:**

First, we retrieved RNA-seq and clinical data of 499 PCa and 52 normal prostate tissue samples from the Cancer Genome Atlas (TCGA). We identified 193 differentially expressed immune-related genes (IRGs) between PCa and normal prostate tissues. Functional enrichment analyses showed that the immune system can participate in PCa initiation. Then, we constructed a correlation network between transcription factors (TFs) and IRGs. We performed univariate and multivariate Cox regression analyses and identified five key prognostic IRGs (S100A2, NOX1, IGHV7-81, AMH, and AGTR1). Finally, a predictive nomogram was established and verified by the C-index.

**Results:**

We successfully constructed and validated an immune-related PCa prediction model. The signature could independently predict PCa patients’ survival. Results showed that high-immune-risk patients were correlated with advanced stage. We also validated the S100A2 expression *in vitro* using PCa and normal prostate tissues. We found that higher S100A2 expressions were related to lower biochemical recurrences. Additionally, higher AMH expressions were related to higher Gleason score, lymph node metastasis and positive rate, and tumor stages, and higher ATGR1 expressions were related to lower PSA value.

**Conclusion:**

Overall, we detected five IRGs (S100A2, NOX1, IGHV7-81, AMH, and AGTR1) that can be used as independent PCa prognostic factors.

## Introduction

According to the latest available data, prostate cancer (PCa) is the most common cancer diagnosed among men and the second leading cause of male cancer deaths in the United States (1, 2). The PCa incidence has recently risen and can be explained by the widespread use of prostate-specific antigen (PSA) tests (3). Studies have identified different PCa risk factors such as genetics, diet, and hormones (4). Radical prostatectomy or radiation are standard primary treatments for localized PCa patients, However, for recurrent- or advanced-staged PCa patients, the standard treatment can be accompanied by therapy intensification (5–7). PCa’s endocrine therapy includes antiandrogen and castration. Recently, gene therapy has become increasingly popular and already showed some clinical achievements (8). However, solid data are still lacking for further investigation of PCa molecular profiles that could reveal novel PCa diagnosis targets and treatments (9–11).

Despite the initial effective responses with androgen suppression therapy (AST), almost all patients progress to metastatic castration-resistant prostate cancer (mCRPC) (12, 13). Currently, docetaxel, abiraterone, cabazitaxel, and Sip-T are approved by the FDA for mCRPC treatment. However, these treatments only provide 2–4 median survival months’ benefits (14–18). The mCRPC patients’ median overall survival (OS) ranges from 13 to 32 months with a 15% 5-year survival rate. Therefore, new mCRPC treatments are urgently required.

In the last decade, immunotherapy has achieved significant milestones. In 2010, the FDA approved the first dendritic cell-based vaccine, Sip-T, for non-symptomatic metastatic PCa treatments (19). In 2011, a CTLA-4 immune checkpoint inhibitor, ipilimumab, was approved for metastatic melanoma treatment (20, 21). From 2014, PD-1/PD-L1immune checkpoint inhibitors were approved for different cancers including lung cancer, kidney cancer, urothelial carcinoma, Hodgkin’s disease, breast cancer, as well as for microsatellite high and deficient solid tumors mismatch repair (22–24). However, Sip-T remains the only immunotherapy approved for PCa treatment. Currently, different clinical trials are evaluating immunotherapeutic agents and their efficacy.

In this study, we utilized TCGA transcriptome data to develop and validate a PCa risk signature with five IRGs: S100A2, NOX1, IGHV7-81, AMH, and AGTR1. We also analyzed the signature correlation with other factors to determine its clinical value.

## Materials and Methods

### Data Collection and Immune-Related Genes

PCa samples’ clinical and transcriptomic data were collected from UCSC-TCGA (https://tcga-data.nci.nih.gov/tcga/). The RNA expression profiles were obtained by RNA-seq, and the log_2_-based transformation was used for normalization. We compared these RNA-seq data with the Immport database. RNA-seq data were standardized using the R language.

### Differentially Expressed Immune-Related Gene Enrichment Analyses

The “Limma” R package was applied to estimate differentially expressed IRGs between PCa and normal samples. Genes exhibiting at least 2-fold changes and corresponding to an adjusted *p* < 0.05 were selected as being differentially expressed. First, a heatmap and a volcano map were used to filter these differentially expressed IRGs. Then, we performed a series of gene functional enrichment analyses, gene ontology (GO) and Kyoto Encyclopedia of Genes and Genomes (KEGG), to detect major biological attributes. The Database for Annotation, Visualization, and Integrated Discovery (DAVID) (https://david.ncifcrf.gov/) was used for functional annotation and to identify GO and KEGG enriched terms. The “GO plot” R package was used to visualize enriched terms.

### Construction and Validation of a Novel Immune-Related PCa Prognostic Signature

To analyze the IRGs ontology terms of signature, gene set enrichment analysis (GSEA) was performed between high- and low-risk groups (https://pypi.org/project/gseapy/). Twenty-two GO gene sets were retrieved from the Molecular Signatures Database (MSigDB) (http://software.broadinstitute.org/gsea/downloads.jsp). A *p*-value < 0.05 was considered statistically significant for enriched gene sets with nominal and false discovery rates (FDR) < 0.25.

To identify key IRGs, univariate and multivariate Cox regression analyses were performed to exclude IRGs with little prognostic value. According to each gene weight in the multivariate Cox regression analysis, the correlation coefficients for the patients’ prognostic prediction model were obtained. Combining differently expressed genes, we established an independent prognostic model. The prognostic index was calculated as follows:

$$\left(\beta_{1}\times \text{gen}{\text{e}}_{1}\text{expression})+(\beta_{2}\times \text{gen}{\text{e}}_{2}\text{expression})+\cdot \cdot \cdot \cdot \cdot +(\beta \text{n}\times \text{gen}{\text{e}}_{\text{n}}\text{expression}\right)$$

Where β corresponds to the correlation coefficient.

### Identification of Differently Expressed Transcription Factors and Construction of a Correlation Network Between TFs and IRGs

We used the Cistrome database (http://www.cistrome.org/) to predict TF targets and extract cancer enhancer profiles. The prediction was performed based on TCGA expression profiles integrative analysis and public ChIP-seq profiles. Then, we identified differently expressed TFs between PCa and normal prostate tissues with the “Limma” R package. We also tested the correlation between differently expressed TFs and IRGs. A correlation coefficient >0.4 with a *p* < 0.01 was considered as a remarkable correlation.

### Immune-Related PCa Prognostic Signature Evaluation

According to our prognostic model, each patient received a risk score. We set the median risk score as the cutoff value to divide PCa patients into a high- and low-risk groups. The Kaplan-Meier (K-M) method was utilized to plot the survival curves, and the log-rank test was performed to assess different survival rates between groups. Receiver operating characteristic (ROC) curves were created with the “survival ROC” R package, and the area under the curve (AUC) was calculated to evaluate model specificity and sensitivity. Patients’ risk score distribution in different risk groups and the survival state program showed that high-risk-score patients had higher death rates. We also showed the number of censored patients and constructed a prognosis-related IRGs heatmap. Finally, a prognostic nomogram was constructed to predict PCa patients’ survival time.

### Correlations Between the Immune-Related Risk Signature and Clinicopathologic Features

Further, to improve the PCa prognostic signature OS prediction accuracy, we integrated age, race, T, N, lymph nodes positive, Gleason score, PSA value, and biochemical recurrence in our evaluations.

### Immunohistochemistry

Ten samples were obtained from patients approved by the local ethics committee of the Zhongda Hospital Affiliated with the Southeastern China University. The antibody against S100A2 was obtained from Abcam. Prostate tissues were fixed with 4% paraformaldehyde, embedded in paraffin, then sectioned. Tissue sections were placed into a box and heated in a microwave oven for antigen retrieval. Then, we blocked the endogenous peroxidase by treating the sections with 3% hydrogen peroxide. Sections were incubated with a primary antibody followed by an appropriate secondary one. Antibody binding was visualized by DAB treatment. The nuclei were stained, and sections were dehydrated. Finally, sections were mounted on glass slides for analysis.

### Quantitative Real-Time PCR Analysis

We used RNA extraction kits (Invitrogen, CA, USA) to extract RNA from prostate tissues. RNA concentrations were measured with a Tecan Sunrise infinite M200 PRO plate-reader (Tecan, Switzerland), and mRNA expressions were normalized with GAPDH. The specific primers used were: S100A2 5¢-ACCGACCCTGAAGCAGAACTC-3¢ and 5¢-CCTCATCTCCCAGCACTCCA-3¢; GAPDH 5¢-CTTTGGTATCGTGGAAGGACTC-3¢ (forward), 5¢-GTAGAGGCAGGGATGATGTTCT-3¢ (reverse). Each experiment was repeated at least three times.

### Western Blotting

Prostate tissues were used for protein extraction, and protein concentration was determined using a BCA Protein Assay Kit (Pierce, USA). First, samples were separated by 10% sodium dodecyl sulfate-polyacrylamide gel electrophoresis (SDS-PAGE), then transferred to polyvinylidene fluoride (PVDF, Millipore, USA) membranes. Membranes were blocked with 5% skimmed milk for 60 min at room temperature, then incubated with S100A2 and GAPDH antibodies overnight at 4°C. Membranes were washed three times with TBST buffer (20 mmol/L Tris-buffered saline and 0.1% Tween 20), then incubated with peroxidase (HRP)-conjugated secondary antibody for 1 h at 37°C. Finally, immunoblotted proteins were analyzed using ImageJ (National Institutes of Health, USA).

### Statistical Analyses

Heatmaps and boxplots were generated with the “Complex Heatmap” and “ggplot2” R package, respectively. We calculated the C-index with the “survcomp” R package. The Student’s t-test was used for paired data comparison, and the ANOVA test was conducted for comparison of more than two scores. Pearson’s χ^2^ tests were performed for categorical variables comparison. Tests were performed using the “stats” R package version 3.5.1. Each experiment was repeated at least three times. Data are presented as means ± standard deviations (SD), and a *p* < 0.05 was considered statistically significant. Statistical and data analyses were conducted with the R (https://www.r-project.org/) and the GraphPad Prism 8.0 software.

## Results

### Differentially Expressed Immune-Related Genes

**Figure 1:**
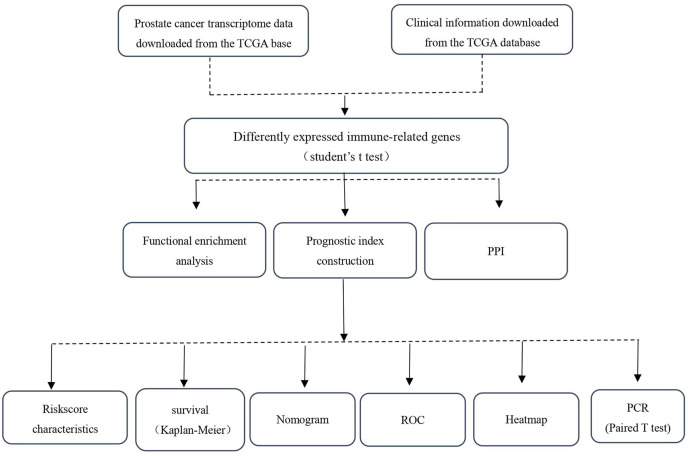
Research workflow.

**Figure 2:**
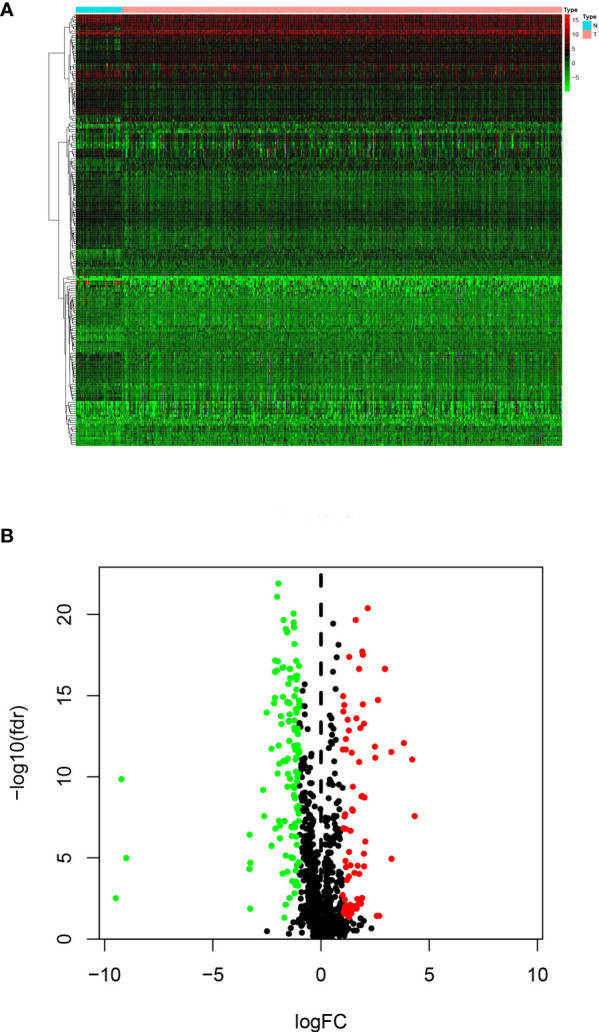
Differentially expressed IRGs: **(A)** Heatmap and **(B)** Volcano map.

RNA-seq and clinical data from 499 PCa and 52 normal samples were retrieved from TCGA. All PCa patients with gene expression data and clinical information were enrolled in the current study (**Figure 1**). Then, we initially screened 193 differentially expressed genes (**Figures 2A, B**).

### Network Analyses of TF-IRG Interactions

**Figure 3:**
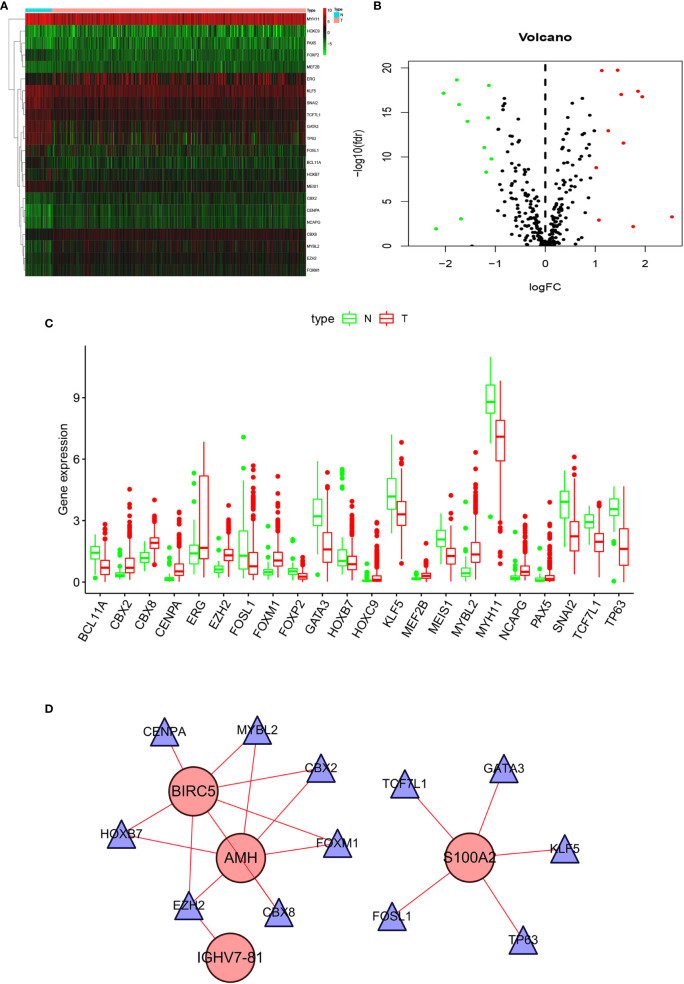
Differentially expressed TFs: **(A)** Heatmap, **(B)** Volcano map, and **(C)** Boxplots. **(D)** The network shows the relationship between TFs and ARGs.

To explore TF and IRG interactions, we extracted 22 differentially expressed TFs between PCa and normal prostate tissues (**Figure 3A**). We detected 11 upregulated and 11 downregulated TFs (**Figures 3B, C**). The TF-IRG interaction network is detailed in **Figure 3D**. TF-IRG interaction network results had no direct influence on this research conclusion. However, the TFs found can direct further researches.

### Functional Annotation of Differentially Expressed IRGs

**Figure 4:**
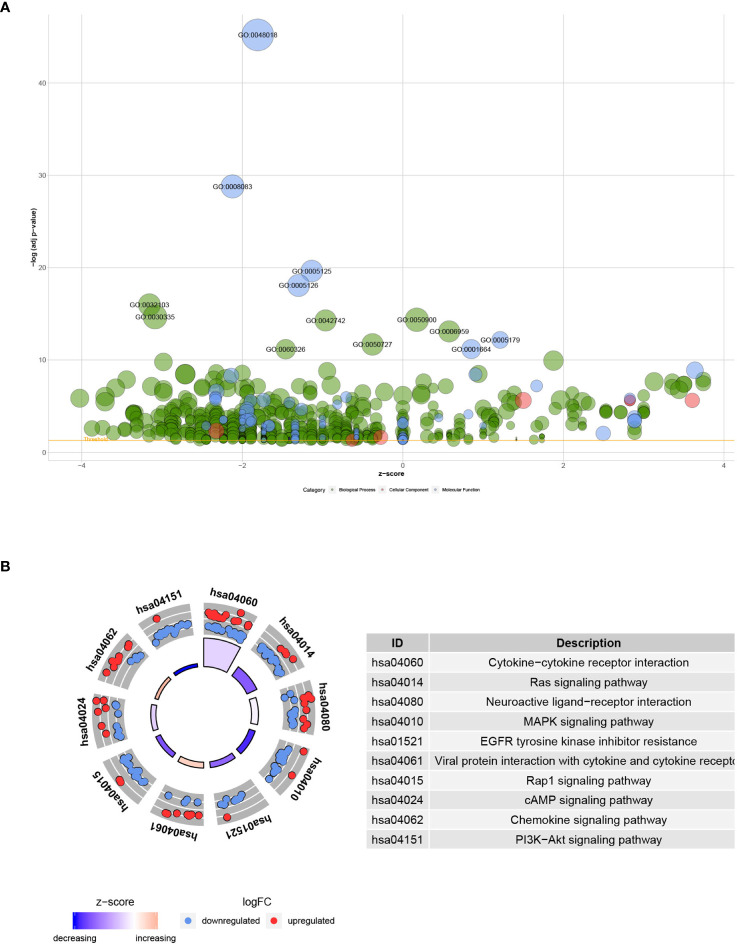
Functional annotation of differentially expressed IRGs: **(A)** Enriched GO terms bubble plot. Green circles correspond to the biological process, red indicates the cellular component, and blue shows the molecular function category. **(B)** KEGG pathways circle diagram. Red circles represent upregulation, and blue ones represent downregulation.

The functional enrichment analysis of the 193 differentially expressed IRGs demonstrated their biological roles. GO functional terms and KEGG enriched pathways are summarized in **Tables 1**, **2**, respectively, and the schematic overview of these results is presented in **Figure 4**. According to DAVID results, the top enriched GO terms for biological processes were as follows: response to external stimulus positive regulation (1.06E-16), cell migration positive regulation (2.19E-15), and leukocyte migration (4.46E-15). For cellular components, the top terms were immunoglobulin circulating complex (2.38E-06), plasma membrane external side (2.38E-06), and immunoglobulin complex (2.38E-06). Based on molecular function, genes were mostly enriched regarding receptor-ligand (6.31E-46), growth factor (1.62E-29), and cytokine (2.33E-20) activities. Besides, in the KEGG pathway enrichment analysis, the differentially expressed IRGs were associated with Cytokine–cytokine receptor interaction (3.86E-16), Ras signaling (6.70E-08), Neuroactive ligand-receptor interaction (5.64E-06), MAPK signaling (1.07E-05), and EGFR tyrosine kinase inhibitor resistance (1.71E-05). Enriched pathways Z-scores < 0 indicated that most cancer pathways were more likely to be decreased (**Figures 4A, B**).

**Table 1:** GO terms function of IRGs for PCa patients.

ONTOLOGY	ID	Description	p value	p. adjust	Count
BP	GO:0032103	Positive regulation of response to external stimulus	3.47E-20	1.06E-16	29
BP	GO:0030335	Positive regulation of cell migration	1.44E-18	2.19E-15	34
BP	GO:0050900	Leukocyte migration	4.38E-18	4.46E-15	33
BP	GO:0042742	Defense response to bacterium	6.88E-18	5.25E-15	27
BP	GO:0006959	Humoral immune response	1.31E-16	8.01E-14	27
BP	GO:0050727	Regulation of inflammatory response	4.10E-15	2.09E-12	28
BP	GO:0060326	Cell chemotaxis	1.58E-14	6.87E-12	23
BP	GO:0002460	Adaptive immune response based on somatic recombination of immune receptors built from immunoglobulin superfamily domains	3.09E-13	1.18E-10	23
BP	GO:0050920	Regulation of chemotaxis	4.80E-13	1.63E-10	19
BP	GO:0030595	Leukocyte chemotaxis	5.10E-12	1.56E-09	18
CC	GO:0042571	Immunoglobulin complex, circulating	2.51E-08	2.38E-06	8
CC	GO:0009897	External side of plasma membrane	3.82E-08	2.38E-06	16
CC	GO:0019814	Immunoglobulin complex	4.13E-08	2.38E-06	8
CC	GO:0072562	Blood microparticle	4.56E-08	2.38E-06	13
CC	GO:0031012	Extracellular matrix	0.0011229	0.004649379	15
CC	GO:0043025	Neuronal cell body	0.00170151	0.023343588	13
CC	GO:0034774	Secretory granule lumen	0.001628514	0.048622789	10
MF	GO:0048018	Receptor ligand activity	1.86E-48	6.31E-46	60
MF	GO:0008083	Growth factor activity	9.51E-32	1.62E-29	32
MF	GO:0005125	Cytokine activity	2.06E-22	2.33E-20	28
MF	GO:0005126	Cytokine receptor binding	1.01E-20	8.55E-19	29
MF	GO:0005179	Hormone activity	9.94E-15	6.76E-13	17
MF	GO:0001664	G-protein coupled receptor binding	1.17E-13	6.65E-12	22
MF	GO:0003823	Antigen binding	2.68E-11	1.30E-09	17
MF	GO:0042379	Chemokine receptor binding	8.61E-11	3.66E-09	11
MF	GO:0070851	Growth factor receptor binding	1.31E-10	4.95E-09	14
MF	GO:0008009	Chemokine activity	1.89E-09	6.43E-08	9

BP, biological processes; CC, cellular components; MF, molecular function.

**Table 2:** KEGG pathway enrichment of IRGs for PCa patients.

ID	Description	p value	p.adjust	Count
hsa04060	Cytokine–cytokine receptor interaction	2.03E-18	3.86E-16	32
hsa04014	Ras signaling pathway	7.05E-10	6.70E-08	20
hsa04080	Neuroactive ligand-receptor interaction	8.91E-08	5.64E-06	21
hsa04010	MAPK signaling pathway	2.25E-07	1.07E-05	19
hsa01521	EGFR tyrosine kinase inhibitor resistance	4.84E-07	1.71E-05	10
hsa04061	Viral protein interaction with cytokine and cytokine receptor	5.40E-07	1.71E-05	11
hsa04015	Rap1 signaling pathway	1.30E-06	3.53E-05	15
hsa04024	cAMP signaling pathway	1.65E-06	3.92E-05	15
hsa04062	Chemokine signaling pathway	1.05E-05	0.000220858	13
hsa04151	PI3K-Akt signaling pathway	1.40E-05	0.000265748	18

### Immune-Related Risk Signature Construction and Validation

**Figure 5:**
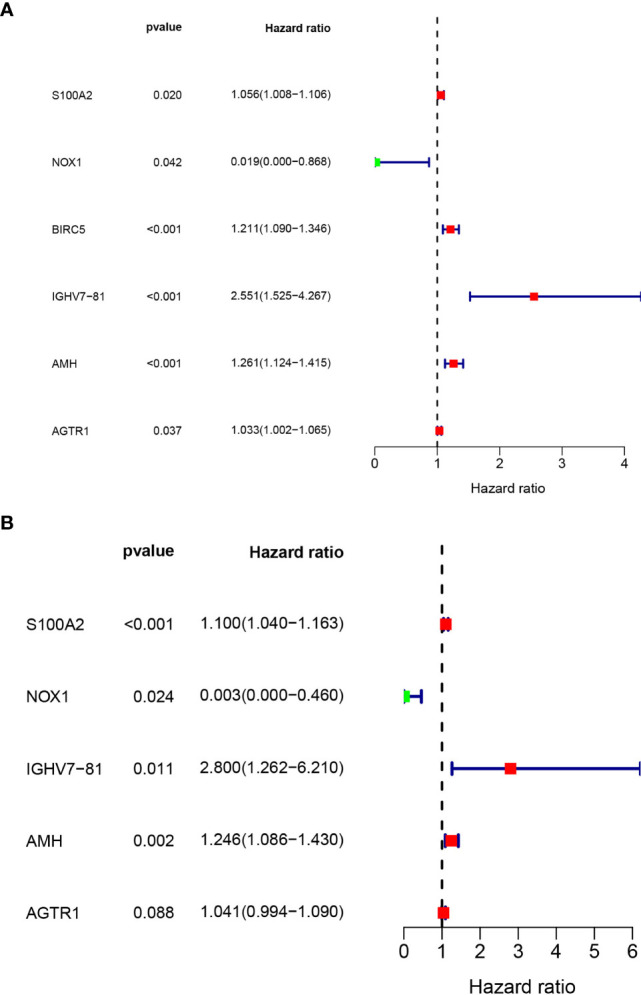
Prognostic IRGs identification: **(A)** Univariate and **(B)** Multivariate Cox regression analyses.

These survival-related genes were subjected to univariate and multivariate Cox regression analyses to remove genes that were not prognostic independent indicators. Then, different prognostic IRGs were obtained. The relationships between the 193 differentially expressed IRGs profiles were obtained from TCGA and resulted in six prognosis-related IRGs (S100A2, NOX1, IGHV7-81, AMH, AGTR1, and BIRC5) (**Figure 5A**). To improve robustness, these IRGs were used in a multivariate Cox regression model, performed by SPSS 24.0 (**Figure 5B**). However, the AGTR1 gene showed no significant prognostic value (*p* > 0.05). Finally, five genes—S100A2, NOX1, IGHV7-81, AGTR1, and AMH—were identified and integrated into the model (**Tables 3**, **4**).

**Table 3:** Univariate Cox regression analysis to filtrate key ARGs for BC patients.

ID	HR	HR.95L	HR.95H	p value
S100A2	1.056105264	1.008492562	1.105965846	0.020380834
NOX1	0.019298037	0.000428891	0.868320058	0.042086476
BIRC5	1.211058502	1.089847975	1.345749799	0.00037223
IGHV7-81	2.550963691	1.525108226	4.266855059	0.000359584
AMH	1.261444149	1.124176792	1.415472506	7.77E-05
AGTR1	1.033184764	1.001946872	1.065396567	0.037148163

**Table 4:** Multivariate Cox regression analysis to filtrate key ARGs for BC patients.

ID	coef	HR	HR.95L	HR.95H	p value
S100A2	0.095097498	1.099766075	1.040001191	1.162965418	0.000850669
NOX1	−5.852129483	0.002873773	1.79E-05	0.460277302	0.023848854
IGHV7-81	1.029487511	2.799630687	1.262231918	6.209581514	0.011310899
AMH	0.220039707	1.24612621	1.085665335	1.430303134	0.001756294
AGTR1	0.039940648	1.040749001	0.994116914	1.089568509	0.087694566

### Evaluation of the Immune-Related PCa Prognostic Signature

**Figure 6:**
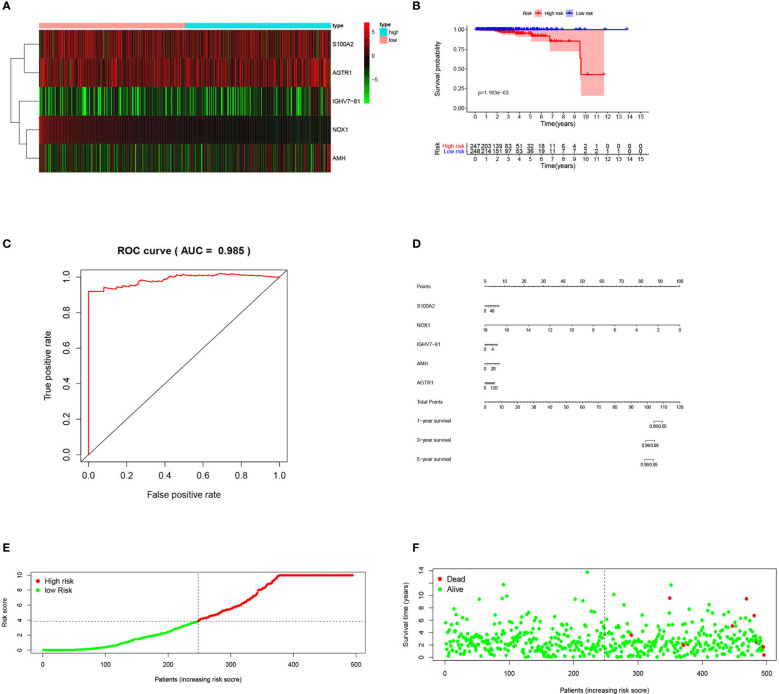
Evaluation of prognostic index based on IRGs for PCa patients: **(A)** Heatmap; **(B)** Kaplan–Meier plot; **(C)** ROC curve; **(D)** Prognostic nomogram; **(E)** Risk score; **(F)** Survstat.

After construction, we validated and evaluated the immune-related PCa prognostic model. K-M analysis results indicated that the high-risk group had a shorter survival time compared to the low-risk group (log-rank test *p-*value = 1.163E−03) (**Figure 6A**). The prognosis-related IRGs heatmap is also presented (**Figure 6B**). To evaluate the immune-related prognostic signature effectiveness, a time-dependent ROC curve analysis was performed using the “survival ROC” R package. The AUC was also calculated to evaluate the model’s specificity and sensitivity. The prognostic signature AUC was 0.985, demonstrating that the prognostic signature is suitable for survival predictions (**Figure 6C**). We also constructed a prognostic nomogram (“rms” R package) to predict patients’ survival rates based on IRGs and the Cox proportional hazard regression model (**Figure 6D**). Finally, the patients’ risk score distribution showed that the high-risk group had higher risk scores, and the survival state program showed that high-risk-score patients had higher death rates (**Figures 6E, F**).

### Combination of the Prognostic Signature and Clinical Parameters

**Figure 7:**
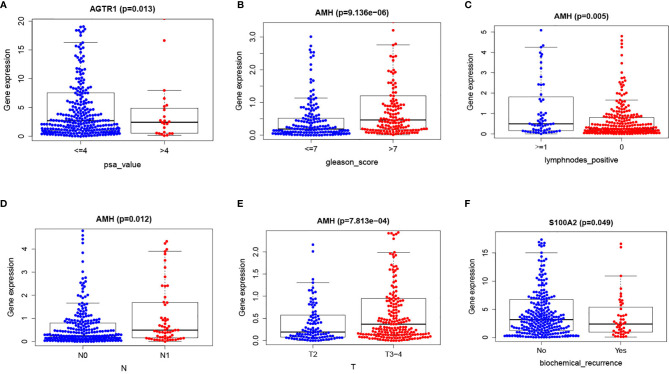
Immune-related risk signature with clinicopathologic features correlations: **(A)** ATGR1 and PSA value; **(B)** AMH and Gleason score; **(C)** AMH and lymph nodes positive; **(D)** AMH and N; **(E)** AMH and T; **(F)** S100A2 and biochemical recurrence.

Results showed that higher S100A2 expressions were related to lower biochemical recurrences. Additionally, higher AMH expressions were related to higher Gleason scores, lymph node metastasis rates, tumor grades, and lower positive lymph nodes. Finally, higher ATGR1 expressions were related to lower PSA values (**Figures 7A–F**, **Table 5**).

**Table 5:** Clinical correlation analysis between these five prognostic IRGs, our established risk score, and clinical features.

ID	Age	Race	T	N	Lymphnodes_positive	Gleason_score	psa_value	Biochemical_recurrence
S100A2	−0.597 (0.551)	0.399 (0.819)	−0.868 (0.386)	−0.864 (0.391)	0.849 (0.399)	−0.305 (0.760)	−0.017 (0.986)	1.993 (0.049)
NOX1	−1.193 (0.235)	0.263 (0.877)	−1.379 (0.169)	−0.154 (0.878)	0.141 (0.888)	−0.861 (0.390)	0.093 (0.927)	−0.086 (0.931)
IGHV7-81	−1.914 (0.058)	2.319 (0.314)	0.021 (0.983)	1.185 (0.238)	−1.024 (0.307)	−1.845 (0.067)	−0.25 (0.804)	−0.812 (0.419)
AMH	−1.736 (0.085)	0.486 (0.784)	−3.391 (7.813e-04)	−2.562 (0.012)	2.903 (0.005)	−4.56 (9.136e-06)	0.063 (0.950)	−1.019 (0.311)
AGTR1	−1.954 (0.053)	0.197 (0.906)	−0.953 (0.341)	1.603 (0.111)	−1.694 (0.092)	1.956 (0.051)	2.573 (0.013)	0.007 (0.994)
riskScore	−0.492 (0.623)	0.21 (0.900)	−0.352 (0.725)	−0.819 (0.416)	0.816 (0.418)	−1.536 (0.127)	1.411 (0.159)	1.528 (0.128)

### Validation of S100A2 as a Prognostic Factor

**Figure 8:**
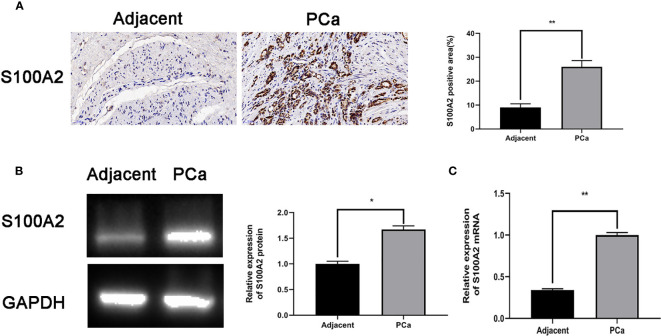
S100A2 validation as a prognostic factor: **(A)** Immunohistochemistry and quantitative analysis in tumor-adjacent and PCa tissues; **(B)** S100A2 mRNA expression in tumor-adjacent and PCa tissues; **(C)** S100A2 protein expression in tumor-adjacent and PCa tissues. *p < 0.05; **p < 0.01.

S100A2 had the lowest *p-*value in our prognostic model. To validate this result, human prostate tissues were used to confirm the S100A2 higher expression. Compared with tumor-adjacent tissues, the S100A2 expression was indeed higher in PCa samples (**Figures 8A–C**).

## Discussion

PCa is the most common malignancy diagnosed among men and is ranked as the second leading cancer-related death cause in US men, becoming the most common cancer affecting men’s health in this country (25). Although the PCa incidence in Asia is much lower than in Europe and the US, it has been recently rising faster than these two other regions (26). However, there are no immunological markers available to predict PCa patients’ survival. Therefore, we obtained PCa and normal samples RNA-seq and clinical data from TCGA that provided effective measures for gene signature construction. We evaluated IRGs’ expression profiles and detected prognostic signatures for PCa patients.

First, we screened 193 differentially expressed IRGs between PCa and non-tumor tissues. Based on these results, we extracted 22 differentially expressed TFs by RNA-seq data analysis to construct a TF-IRG interaction network. Considering that these genes might be deeply involved in PCa initiation, we performed GO and KEGG enrichment analyses. To identify key IRGs in the prognostic signature, univariate and multivariate Cox regression analyses were performed. These analyses showed five key IRGs (S100A2, NOX1, IGHV7-81, AMH, and AGTR1) that could be used as a PCa prognostic signature in the TCGA database. Then, we validated and evaluated this model and linked it to clinical factors.

At present, several studies have built prediction models using different databases. For example, Wang et al. analyzed 411 BC patients and 19 non-tumor samples RNA-seq data from TCGA and obtained an individualized autophagy-clinical prognostic index with prediction value (27). Also, An et al. analyzed the RNA-seq data of 117 serous ovarian cancer and 52 normal ovarian tissues from GEO datasets (28). This study is the first to build an immune predictive model for PCa and to correlate it with clinicopathological factors. We also internally validated our results. Due to the lack of sufficient clinical samples, we only validated the S100A2 expression between PCa and normal tissues.

S100A2 (S100 Calcium Binding Protein A2) has calcium-binding motifs and is involved in the regulation of different cellular processes such as cell cycle progression and differentiation. Reports have indicated that S100A2 downregulation in PCa is related to a biologic aggressiveness increase (29). A transcriptional cross-talk may exist between S100A2 and p53, an immune-related pathway key gene (30). Also, an age-related effect was detected for S100A2 methylation levels in BPH patients (31). Altogether, this might explain the differences in S100A2 expression levels and immune-related behaviors.

The Nox1 (NADPH Oxidase 1) gene encodes an enzyme responsible for oxygen single electron transfer catalysis to produce superoxide or hydrogen peroxide. This function has already been verified in human PCa, and elevated Nox1/H2O2 levels are associated with malignant transformation and tumorigenicity increases in PCa animal models (32). These effects might be caused by the immune cell-mediated inflammation triggered by the release of reactive oxygen species (ROS) induced by Nox1 (33).

The AGTR1 (Angiotensin II Receptor Type 1) gene encodes a type 1 receptor that can mediate major angiotensin II major cardiovascular effects. AGTR1 plays a key role in effective androgen-independent DU145 cell proliferation and metastasis inhibition *in vitro*, *via* AGTR1-dependent apoptotic pathways (34). AGTR1 can also promote lymph node metastasis by chemokine CXCR4/SDF-1α increases, presenting a cancer-promoting effect. Its underlying mechanism may depend on FAK/RhoA pathway activation (35).

IGHV7-81 (Immunoglobulin Heavy Variable 7-81) and AMH (Anti-Mullerian Hormone) relations with PCa were not previously reported. However, our results showed that they could be involved in specific tumor immunity or affect PCa progression. Therefore, a novel model based on them can be crucial to high-risk patients’ assessment.

The BRCA2 gene is related to the inhibition of malignant tumor occurrence (36). BRCA2 mutations are reported in approximately 5% of progressive PCa patients, presenting a higher chance of advanced-stage tumors (37, 38). Also, patients with germline BRCA2 gene mutations and diagnosed with localized PCa had reduced cancer-specific survival compared to non-carriers (39). Recent studies have reported that nearly 25% of advanced PCa patients with DNA damage repair defects were also associated with progressive BRCA2 deficiencies (40). Additionally, BRCA2 can trigger immune responses linked to PCa. However, BRCA2 was not present in our immune-related PCa gene set.

TF-IRG interaction network included six upregulated TFs (CBX2, CBX8, CEPNA, EZH2, FOXM1, MYBL2) and six downregulated TFs (FOSL1, GATA3, HOXB7, KLF5, TCF7L1, TP63). CBX2 inhibition could induce cancer cell death, and CBX2 was positioned as a drug target in lethal castration-resistant PCa (CRPC) (41). The CBX8 expression in esophageal squamous cell carcinoma was significantly higher than that in paracancer tissues, and the increase extent was related to the TNM stage (42). CENPA was significantly overexpressed in PCa patients, and overexpression correlated with progression (43). Dysregulated expression of EZH2 was involved in the PCa progression, as well as being a marker that distinguished indolent PCa from lethal PCa (44). In summary, 12 TFs have been reported to be associated with PCa, except for CBX8. However, the literature for 12 TFs was limited, and no relevant literature has made a summary of 12 TFs. In this research, the relationship between TFs and IRGs was obtained.

Overall, we developed an IRGs model that can independently predict the PCa patients’ overall survival, showing that targeted IRG therapies might be promising for PCa treatment. Further investigations regarding IRGs’ molecular mechanisms would demonstrate how they affect PCa survival and provide new treatment suggestions. Therefore, our signature containing five IRGs can predict PCa patients’ OS and guide novel therapeutic approaches.

## Conclusion

Five IRGs—S100A2, NOX1, IGHV7-81, AMH, AGTR1—were filtrated and utilized to establish a novel immune-related signature. The IRGs-based signature was successfully established and internally verified for PCa patients’ OS prediction. A TF-IRG interaction network was also constructed and can provide directions for future researches. Our established signature showed excellent PCa predictive efficacy and was significantly associated with clinical parameters. We believe that our results might help clinicians predict PCa patients’ survival. However, only the S100A2 expression was verified in human bladder tissues and showed significance between bladder tumor and normal tissues. Further researches are required to verify our findings for the other IRGs.

## Data Availability Statement

The datasets presented in this study can be found in online repositories. The names of the repository/repositories can be found here: https://tcga-data.nci.nih.gov/tcga/.

## Ethics Statement

The studies involving human participants were reviewed and approved by Zhongda Hospital Affiliated to Southeastern China University. The patients/participants provided their written informed consent to participate in this study.

## Author Contributions

MC and SC designed the study. HC, YW, and CL conducted the study and maintained the data. TW analyzed the data and made the figures. All authors drafted and revised the paper. All authors contributed to the article and approved the submitted version.

## Funding

This work was supported by the Key Program Project of Jiangsu Province [grant number BE2019751] and the National Natural Science Foundation of China [grant number 82070773].

## Conflict of Interest

The authors declare that the research was conducted in the absence of any commercial or financial relationships that could be construed as a potential conflict of interest.

## Publisher’s Note

All claims expressed in this article are solely those of the authors and do not necessarily represent those of their affiliated organizations, or those of the publisher, the editors and the reviewers. Any product that may be evaluated in this article, or claim that may be made by its manufacturer, is not guaranteed or endorsed by the publisher.

## Abbreviations

PCa, Prostate cancer; IRG, immune-related gene; TF, transcription factor; AST, Androgen suppression therapy; mCRPC, metastatic castration-resistant prostate cancer; OS, overall survival; GO, gene ontology; ROC, receiver operating characteristic curves; AUC, area under the curve.
